# Cardiac Magnetic Resonance Imaging in Lyme Carditis—A Case Series and Review of Literature

**DOI:** 10.3390/jcdd12010002

**Published:** 2024-12-25

**Authors:** Matthew Kaczynski, Suhani Desai, Brian Osorio, Edward Hulten, Saurabh Agarwal, Michael K. Atalay, Yash Patel

**Affiliations:** 1 Division of Cardiology, Department of Medicine, The Warren Alpert Medical School of Brown University, Providence, RI 02903, USA; matthew_kaczynski@brown.edu (M.K.); suhanimdesai@gmail.com (S.D.); brianosorio820@gmail.com (B.O.); ehulten@brownhealth.org (E.H.); 2 Department of Diagnostic Imaging, The Warren Alpert Medical School of Brown University, Providence, RI 02903, USA; sagarwal18@gmail.com (S.A.); atalay_99@yahoo.com (M.K.A.)

**Keywords:** *Borrelia*, cardiac, Lyme carditis, Lyme disease, MRI, myocarditis

## Abstract

Lyme carditis is an uncommon but potentially fatal manifestation of early disseminated Lyme disease. Timely diagnosis poses a clinical challenge due to the highly variable and non-specific symptomatology that can be easily overlooked, as well as the limited availability of specific and non-invasive diagnostic tests for assessing cardiac involvement. While cardiac magnetic resonance (CMR) imaging is the standard imaging modality for diagnosing various etiologies of cardiomyopathy, its application in Lyme carditis remains understudied. In this study, we present two cases of CMR-proven Lyme carditis and provide a comprehensive review of the existing literature on the use of CMR in this condition.

## 1. Introduction

Lyme disease (LD), transmitted by the bite of the infected *Ixodes* tick, is the most prevalent vector-borne disease in North America and Europe [1]. While LD primarily affects the skin, joints, and nervous system, cardiac involvement—reported in an estimated 1–10% of LD cases—can have significant implications [2,3,4]. Lyme carditis (LC) is a rare but potentially fatal manifestation of early disseminated LD, caused by the gram-negative spirochete *Borrelia burgdorferi* in North America, as well as *B. afzelii*, *B. garinii*, and *B. spielmanii* in Europe. Epidemiological studies indicate that the incidence of LC varies with season, peaking in the summer and early winter months when tick activity is highest [5]. Young adults and adolescents are most affected, with reports of a 3:1 male-to-female predominance [5]. Moreover, amidst global climate and environmental changes, the incidences of both LD and LC are expected to increase [6,7,8,9,10].

The pathophysiology of LC involves direct infection of cardiac tissue by the spirochete, inducing an inflammatory response [11]. The infection and ensuing inflammation may affect the conduction system of the heart—particularly the AV (atrioventricular) node—resulting in various stages of heart block [11]. Histopathological examinations have shown lymphocytic infiltration in cardiac tissue, indicating an immune-mediated response [12,13]. The clinical presentation may involve a characteristic erythema chronicum migrans (ECM) rash, arthralgias, a non-specific flu-like illness, palpitations, syncope or presyncope, chest pain, and conduction disturbances centralized to the AV node [3,13,14]. These conduction disturbances can lead to various degrees of heart block including first-degree AV block, second-degree AV block, or complete heart block, which in severe cases or in the absence of antibiotic treatment, can necessitate temporary or permanent pace-making to resolve slow or abnormal heart conduction [3,13,14,15].

Timely diagnosis of LC demands a thorough history-taking and comprehensive physical examination, focusing on cardiac symptoms and the presence of LD indicators such as ECM rash, recent tick bites, or travel to an LD endemic region [4,16]. Serology testing including enzyme-linked immunosorbent assay (ELISA) followed by confirmatory Western blot is the standard approach for diagnosing *Borrelia* infection [16]. Electrocardiogram (ECG) is essential for identifying and grading possible heart block as well as assessing overall cardiac function. In some cases, echocardiography may be performed to evaluate cardiac structure and function, especially if other cardiac conditions are suspected. Despite these conventional tests, LC can be a challenging diagnosis in the absence of high clinical suspicion, owing in part to a highly variable symptomatology and a limited repertoire of non-invasive and specific tests to confirm or rule out cardiac involvement by the multisystem infection [4].

Cardiac magnetic resonance (CMR), as endorsed by international guidelines, is a highly sensitive and non-invasive diagnostic tool capable of identifying the presence of active, sub-acute, or chronic myocardial inflammation [17,18]. CMR confers several advantages over conventional imaging techniques, such as echocardiography or computed tomography. It provides detailed anatomical and functional information, allowing for the assessment of myocardial edema, fibrosis, and perfusion abnormalities. The ability to visualize the heart in multiple planes and to quantify cardiac function non-invasively makes CMR particularly valuable. Moreover, specific CMR techniques, including T2 sequences and late gadolinium enhancement (LGE) sequences, can help differentiate between active inflammation and scar formation, thereby informing prognosis and guiding treatment decisions [19]. Recent advancements in CMR technology have further enhanced its diagnostic capabilities. For instance, the use of T1 and T2 mapping has improved the sensitivity and specificity for detecting myocardial inflammation, allowing for earlier and more accurate diagnosis of myocarditis [20,21,22]. Given the potential for rapid clinical deterioration in affected patients, timely recognition of myocarditis is crucial. As such, CMR is increasingly recognized as a first-line imaging modality in the workup of suspected myocarditis, facilitating better patient management and improving outcomes.

Notwithstanding, the utility of CMR with respect to LC is understudied. Herein, we report two cases of LC proven by CMR. In addition, we provide a comprehensive review of the literature on the application of CMR in the setting of LC, with a particular focus on the characteristic imaging findings, diagnostic and prognostic utility, and ability to evaluate long-term persistence or resolution of myocardial inflammation.

## 2. Case Series

### 2.1. Case 1

**Figure 1:**
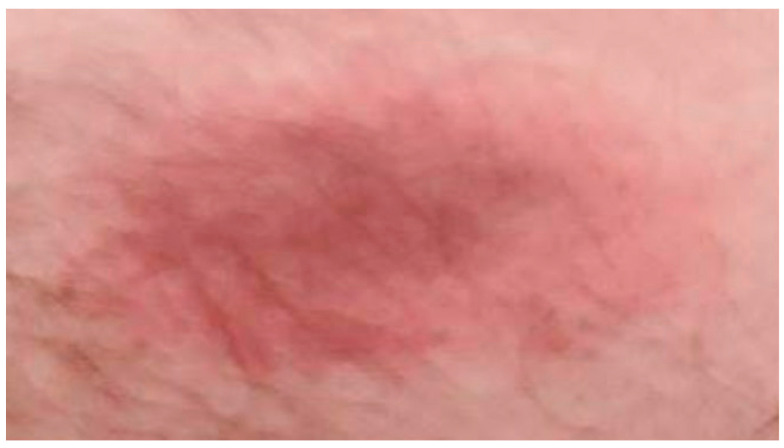
Erythema chronicum migrans rash located on the patient’s right abdomen. Image obtained one week prior to emergency department presentation at an urgent care center.

A 34-year-old male without significant past medical history presented to the emergency department with acute onset chest discomfort that had woken him from his sleep the previous night. One week prior, he was diagnosed with LD at an urgent care center in the setting of myalgias, mild fatigue, intermittent fevers, and an abdominal rash and had initiated treatment with oral doxycycline (Figure 1). Initial ECG demonstrated sinus bradycardia without other ischemic changes or heart block. High-sensitivity troponin (605 ng/L) and inflammatory markers (C-reactive protein, 60.5 mg/L; erythrocyte sedimentation rate, 29 mm/hour) were elevated, and the patient was started on intravenous ceftriaxone and systemic heparin before being transferred to a tertiary care center intensive care unit for further evaluation of possible LC versus acute coronary syndrome.

**Figure 2:**
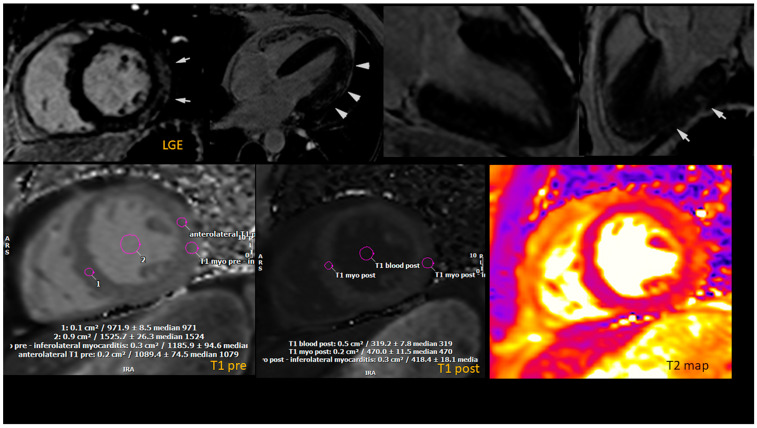
Cardiac magnetic resonance imaging findings. Top images demonstrate the presence of late gadolinium enhancement in lateral segments (white arrows). Bottom images demonstrate corresponding acute inflammation in basal lateral segments.

**Figure 3:**
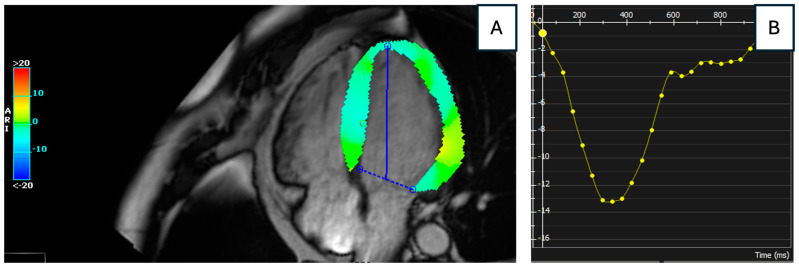
Mildly reduced left ventricular global longitudinal strain measured by cardiac magnetic resonance imaging. (**A**) depicts cardiac magnetic resonance feature tracking global longitudinal strain in four chamber view; (**B**) depicts left ventricular global longitudinal strain (y-axis) over time during cardiac cycle (x-axis). The peak negative value (–13.2%) indicates a reduced maximal degree of myocardial shortening during systole, suggesting impaired left ventricular systolic function.

Following the transfer, troponin levels continued to rise (5672 ng/L). Left heart catheterization and coronary angiogram revealed normal coronary arteries, while echocardiography showed normal biventricular ejection fraction. Lyme serologies collected at admission returned positive for IgM anti-*Borrelia* antibodies. On the fourth day of hospitalization, CMR revealed normal biventricular size and function, with patchy LGE in a sub-epicardial to mid-myocardial distribution in the basal to mid-anterolateral and basal to mid-inferolateral segments, and a subepicardial linear LGE band in the apical lateral segment with an associated T2 signal increase (Figure 2). In addition, left ventricular (LV) global longitudinal strain (GLS) measured by CMR was mildly reduced to –13.2% (Figure 3).

Taken together, these findings conclusively ruled out myocardial ischemia, while CMR confirmed acute myocardial inflammation, supporting a diagnosis of LC. Following clinical improvement with four days of intravenous ceftriaxone, the patient was discharged with instructions to complete a 21-day course of doxycycline, without the need for permanent pacemaker placement.

### 2.2. Case 2

**Figure 4:**
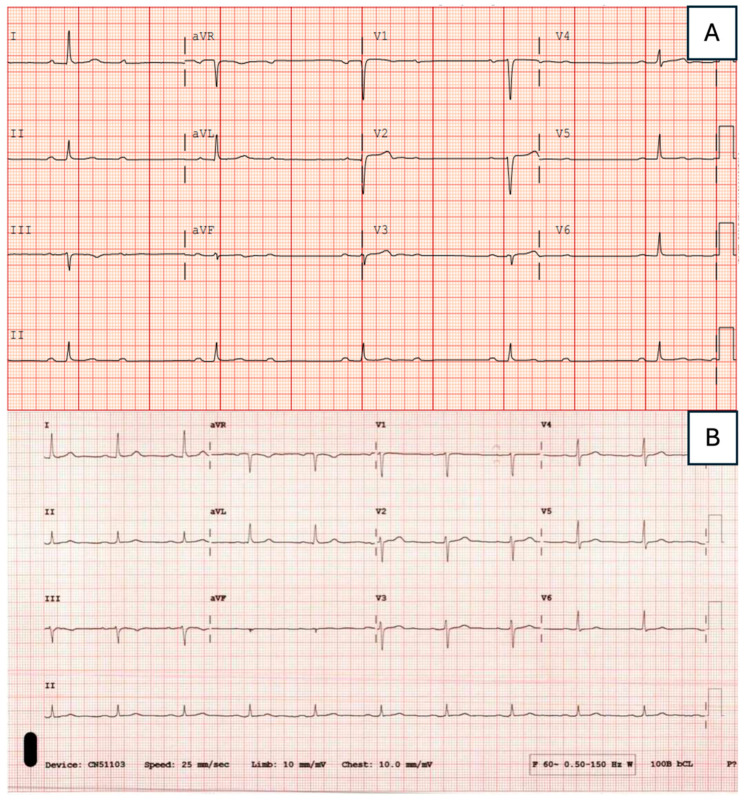
Electrocardiogram findings in Lyme carditis. The baseline ECG (**A**) was performed at presentation to the emergency department and shows 2:1 AV block (Lead II). Follow-up ECG (**B**) was performed after a three-week course of oral doxycycline and demonstrates resolution to normal sinus rhythm with borderline first-degree AV block.

In a separate case, a 62-year-old male with a past medical history of hypertension on once-daily 50 mg losartan and active tobacco use presented to the emergency department with a chief complaint of dizziness, vision changes, and shortness of breath that had begun the previous day. Baseline ECG showed second-degree Mobitz Type II AV block and bradycardia (Figure 4A), and high-sensitivity troponin testing was negative. With deeper questioning of the patient’s medical history, the patient recalled being diagnosed with LD several years prior, with an additional recent tick bite three months before this admission. On the second day of hospitalization, Lyme serologies returned positive for IgM and IgG anti-*Borrelia* antibodies, and the patient was initiated on intravenous ceftriaxone.

**Figure 5:**
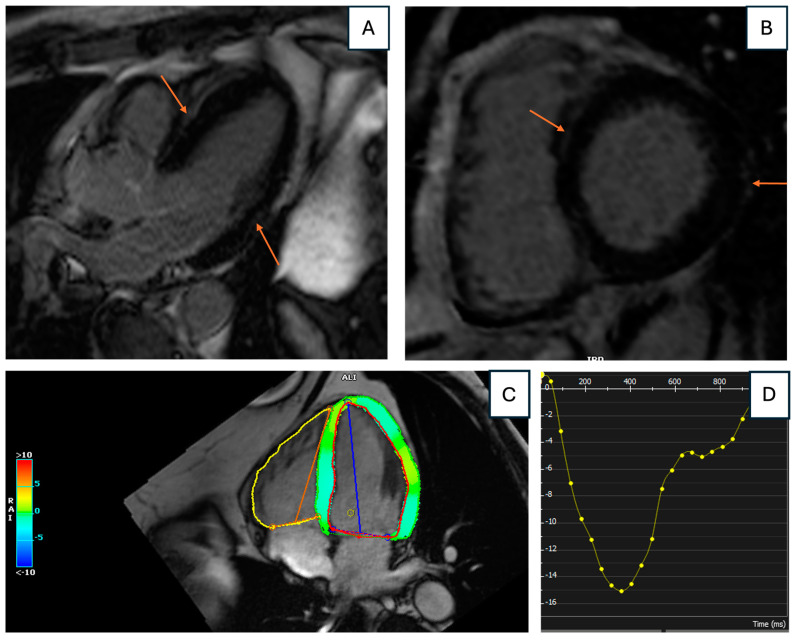
(**A**) (long-axis view) and (**B**) (short-axis view) demonstrate cardiac magnetic resonance imaging with subepicardial late gadolinium enhancement (orange arrows) in the basal anteroseptal and inferolateral segments. (**C**) depicts cardiac magnetic resonance feature tracking global longitudinal strain in four chamber view; (**D**) depicts left ventricular global longitudinal strain (y-axis) over time during cardiac cycle (x-axis). The peak negative value (–15.0%) indicates a reduced maximal degree of myocardial shortening during systole, suggesting impaired left ventricular systolic function.

On the next day, echocardiogram showed normal biventricular size and function. CMR was performed on day four of hospitalization and demonstrated faint subepicardial LGE in the basal anterior septum, as well as the subepicardial mid and apical lateral segments (Figure 5A,B). Additionally, an increased T2 signal was noted over the mid and apical lateral segments, consistent with acute LC. CMR also revealed mildly reduced LV GLS (–15.0%) (Figure 5C,D). Repeat ECG showed improvement in the AV block from second-degree Mobitz Type II to Type I (Wenckebach), and pacemaker implantation was deferred. Once deemed medically stable, the patient was discharged on hospital day five to complete three weeks of oral doxycycline. At an outpatient follow-up visit three weeks post-discharge, heart block had resolved to first-degree AV block without any reported cardiac symptoms (Figure 4B).

In both cases, CMR played an integral role in definitively ruling out ischemic causes of cardiomyopathy and confirming the clinical suspicion of LC. Importantly, in both instances, confirmation of a reversible cause of conduction disturbance allowed both patients to recover without the need for pacemaker implantation or alternative invasive testing techniques such as endomyocardial biopsy.

## 3. Review of Literature

### 3.1. Pathogenesis

**Figure 6:**
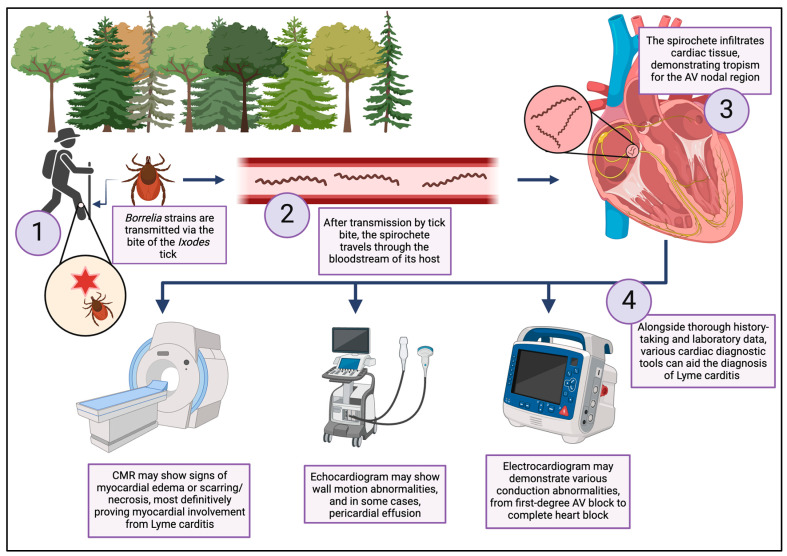
Lyme carditis pathogenesis and the diagnostic role of cardiac imaging techniques. After entering the human host through the bite of the Ixodes tick, *Borrelia* spirochetes demonstrate tropism for cardiac tissue in the AV nodal region, precipitating characteristic conduction disturbances and myocarditis. Cardiac diagnostic modalities, including electrocardiogram, echocardiogram, and cardiac magnetic resonance imaging, can help characterize the extent of cardiac involvement and inform clinical decision-making. Image created in BioRender [24].

The pathogenesis of LC is a multi-mechanistic process (Figure 6). The *Borrelia* spirochete is transmitted to humans in saliva from the bite of the Ixodes scapularis and I. pacificus ticks in eastern and western North America, respectively, or the I. ricinus tick in Europe [4,11,13]. After entering the human host, the bacterium disseminates through the circulatory and lymphatic systems before infiltrating and colonizing cardiac tissue. Myocardial injury ensues as a result of the local inflammatory response, which is marked by the infiltration of lymphocytes, macrophages, and neutrophils into the cardiac tissue and associated edema [11]. In addition, molecular mimicry between *Borrelia* antigens and cardiac tissue may incite an autoimmune response [23], contributing further to the localized inflammation and conduction abnormalities [12].

### 3.2. Clinical Presentation

The clinical presentation of LC is highly variable and may entail a myriad of non-specific symptoms that can be easily overlooked or attributed to more common etiologies including acute coronary syndrome, medication misuse, or other infectious diseases [5,11,25]. Initial signs following the tick bite can include ECM rash along with constitutional symptoms such as fever, chills, and malaise. Cardiac symptoms typically manifest weeks to months after the initial contraction of LD, in the early disseminated stage. Symptoms of chest pain, palpitations, lightheadedness, or syncope may be indicative of AV block or inflammation of the myocardium or pericardium. The characteristic ensuing cardiac pathology is blockage of AV nodal conduction, which can progress from first-degree to complete third-degree block. First-degree AV block consists of a delay between the signaling in the sinoatrial node and ventricles—most often asymptomatic and only notable on ECG. Further progression to second-degree AV block can result in intermittent and symptomatic bradycardia. Low-grade (Mobitz Type I) second-degree heart block often resolves over time, while high-grade (Mobitz Type II) blockages, when permanent, require pacemaker implantation. Third-degree or complete AV block requires pacemaker implantation and may precede hemodynamic instability. In the acute stages of infection, AV block, including advanced high-degree AV block, can be reversible if treated with timely and targeted antimicrobial therapy—without the need for pacemaker implantation. Therefore, rapid diagnosis and tailored therapy are of utmost importance.

### 3.3. Diagnosis of Lyme Carditis

#### 3.3.1. Clinical Tools

The Suspicious Index in Lyme Carditis (SILC) score is a diagnostic tool that evaluates the likelihood that a heart block is secondary to LC [26]. The criteria are based on a 12-point scoring system that includes five variables: constitutional symptoms (i.e., fever, malaise, arthralgia, dyspnea) (2 points), outdoor activity or LD endemic area (1 point), male sex (1 point), tick bite (3 points), age less than 50 years (1 point), and ECM rash (4 points). A score of 7 or higher confers a high pretest probability for LC.

#### 3.3.2. Electrocardiogram

On ECG, LC classically presents with various conduction abnormalities [4,27,28,29]. These abnormalities can progressively worsen in severity as the infection goes untreated, while they tend to revert to normal sinus rhythm with antibiotic therapy. First-degree AV block, seen as a prolonged PR interval (>200 ms), is the most common presentation on ECG. Depending on the disease progression at the time of testing, an initial ECG may also show high-degree AV blocks such as Mobitz Type I, Type II, or complete heart block. Importantly, the AV block in Lyme carditis tends to intermittently fluctuate from one degree to another as it either improves or worsens based on the clinical course. Bundle branch blocks may also be the presenting ECG finding in LC, often seen as wide complexes with characteristic bundle branch patterns such as wide, high-amplitude R and S waves in septal and lateral precordial leads [30,31]. Junctional and ventricular escape rhythms usually present within the context of complete heart block. Although less common, non-heart block arrhythmias such as atrial fibrillation [30,31,32,33,34,35,36] or, in extreme cases, ventricular tachycardia or fibrillation [37,38,39,40,41,42] have also been described in patients with LC.

#### 3.3.3. Echocardiography

Echocardiography can be an accessible and non-invasive tool in the diagnosis of LC. Echocardiographic findings in this condition may include wall motion abnormalities, reduced cardiac contractility, valvular deformities, structural heart changes such as dilated or hypertrophic cardiomyopathy (HCM), or pericardial effusion [13,34,35,43,44]. Although non-specific, these findings alongside additional cardiac tests may help differentiate LC from similarly appearing ischemic and non-ischemic cardiomyopathies (i.e., focal or regional wall motion abnormalities that correlate with areas of infarction in acute coronary syndrome vs. diffuse or global hypokinesia corresponding with immune-mediated inflammatory processes) [45,46,47]. Importantly, several cases of LC that were later proven by CMR featured normal echocardiographic findings at initial presentation, underscoring the limited sensitivity of this imaging modality with respect to LC [40,48,49,50].

#### 3.3.4. Cardiac Magnetic Resonance Imaging

CMR serves as the primary imaging test for diagnosis and prognostication of myocarditis and other non-ischemic cardiomyopathies [51]. CMR provides a non-invasive means of assessing myocardial structure and function, allowing for the quantification of ventricular volumes, contractility, and mass, with superior accuracy than with echocardiography [51]. In addition, CMR uniquely enables the assessment of myocardial inflammation, with the ability to distinguish tissue edema, hyperemia, and necrosis or scarring through the detection of LGE and parametric mapping techniques (T1, T2 and T2* mapping, and extracellular volume measurements) [20,21,22]. Furthermore, CMR allows for the characterization of the intramural pattern (subepicardial, mid-myocardial, subendocardial, transmural, etc.), anatomic location (apical, basal, interventricular septal, etc.), and distribution (focal vs. diffuse) of myocardial inflammation and fibrosis.

Through the collective interpretation of these findings, CMR is capable of prognosticating transient causes of ventricular dysfunction, including myocarditis. By detecting differences in ventricular wall motion patterns, edema and LGE distributions, and cardiac structural alterations, CMR can help assess the extent of myocardial injury, which is critical for stratifying risk and guiding treatment decisions [52]. For instance, in the setting of acute myocarditis, the presence of LGE on CMR may portend a higher likelihood for decreased LV function or progression to dilated cardiomyopathy as well as future mortality. Specifically, the identification of LGE in the anteroseptal region or in a patchy distribution may predict poorer outcomes compared to alternative locations and distributions [19,53]. On the other hand, the absence of LGE may predict improved long-term outcomes, even in the presence of lower ejection fraction or structural changes [53]. Based on these prognostic indicators, medical management can be tailored in the short-term and long-term to incorporate more intensive treatments, increased monitoring, and in some cases, advanced cardiac support or cardiac transplantation.

Additionally, alongside thorough clinical evaluation, CMR enables the differentiation of similarly appearing cardiomyopathies, each carrying unique pathophysiologic origins, treatment regimens, and prognoses. For example, HCM and infiltrative diseases leading to ventricular hypertrophy (i.e., cardiac amyloidosis, sarcoidosis, and LC) can share phenotypic features; however, subtle differences on CMR—based on morphologic changes and inflammatory patterns—can provide critical insights that help inform accurate diagnosis [20,54]. In HCM, LGE may be focal to hypertrophied segments, and edema may be restricted to fibrotic areas. Morphologically, HCM may be associated with LV outflow tract obstruction, along with valvular abnormalities. By contrast, infiltrative causes may present with diffuse or patchy regions of signal enhancement and, in some cases, pericardial effusion. Sarcoidosis can reveal granulomas and hilar lymphadenopathy, while LC may feature inflammation localized to the AV nodal region or global wall motion changes, further distinguishing these etiologies.

The Lake Louise criteria (LLC) are a clinical tool that can aid in the CMR-driven diagnosis of LC. The LLC focus on three diagnostic myocardial targets: edema, hyperemia, and scarring or necrosis. These targets are derived from the evaluation of signal intensity in T1- and T2-weighted early gadolinium and late gadolinium enhancement CMR images [20]. Diagnostic criteria are met when two of the three targets are positive. The LLC also include supportive criteria such as evidence of pericardial effusion and systolic wall motion abnormality [20]. The updated criteria from 2018 have been reported to have a sensitivity of up to 87% and a specificity of up to 96% [55].

Several reports have demonstrated the utilization of CMR to confirm or rule out myocardial involvement in the setting of LC, with 24 CMR-proven LC cases included in this review (Table 1).

Prior studies have described distinct inflammatory patterns by CMR based on the etiology of myocarditis (i.e., viral, eosinophilic, giant cell, etc.) [17,67], though discussion with respect to LC is limited. The goals of CMR in patients with LC include identifying the presence of myocardial edema, identifying the pattern and extent of LGE, excluding other causes of cardiomyopathy, and performing a comprehensive evaluation of LV function [18,20]. Like most other myocarditis etiologies, the majority of included LC cases featured inflammation localized to the subepicardium and mid-myocardium—sparing the subendocardium, a location more commonly associated with ischemic causes of cardiomyopathy [19].

Notably, several reports have described CMR evidence of myocardial inflammation at the location of the AV node. For example, Rojas-Salvador reported the case of a previously healthy 28-year-old male who presented three weeks after a hiking trip in Minnesota, USA, with profound bradycardia (31 bpm) and complete AV block with junctional escape [65]. After the placement of a temporary pacemaker, CMR revealed focal LGE in the basal anteroseptal region near the AV node. Similarly, Inboriboon described a 20-year-old male with two weeks of arthralgias, rashes suspicious for ECM, and a short episode of palpitations [61]. Multiple ECG readings revealed a stepwise progression from first-degree AV block to Type I second-degree AV block to Type II second-degree AV block. CMR showed an increased T2-weighted signal localized to the septum and anterior wall, with an additional focus of LGE in the apical septum, suggestive of inflammation and edema involving the AV node. Altogether, both cases support the established tropism of the spirochete for the AV nodal region and provide a clear pathophysiological link between *Borrelia* infection and its associated cardiac conduction abnormalities as detected by CMR. Additionally, both the diagnosis of LC and the characterization of cardiac involvement were aided by CMR, enabling targeted therapy without the need for permanent pacemaker implantation.

Parametric mapping techniques, such as T1 and T2 mapping, confer additional advantages over the detection of gadolinium enhancement alone in the CMR-supported diagnosis of myocarditis, particularly with respect to the identification of edema in the acute inflammatory phase [21,22]. Kaldas et al. reported the complex case of a 72-year-old male with a known tick-bite, ECM rash, and positive *Borrelia* IgM antibodies who initiated LC treatment with the standard of care, doxycycline, prior to subsequent ventricular tachycardia (VT) arrest 20 days later [41]. Left heart catheterization showed severe stenosis of the left anterior descending artery, though without plaque rupture. It was suspected by the clinical team that coronary artery disease was not the sole cause of the patient’s arrest, and thus, CMR was pursued, which demonstrated multiple patches of mid-myocardial LGE with T2 edema, consistent with acute Lyme-induced myocarditis. Once stabilized, the patient initiated steroid treatment for the component of myocarditis, ceftriaxone for the persisting Lyme infection, and implantable cardioverter-defibrillator placement for secondary prevention of VT. In this case, CMR proved essential to the clinical decision-making process, elucidating the contribution of active LC to the patient’s nearly fatal arrhythmia and informing a more directed treatment plan. In a separate case reported by Ventejou and colleagues, CMR with T2-mapping demonstrated acute myocardial edema in a 64-year-old male with rare LC-associated atrial fibrillation and first-degree AV block in the absence of LGE. Importantly, follow-up CMRs revealed the persistence of myocardial edema, prompting a prolonged treatment course with doxycycline to achieve symptom resolution [36].

### 3.4. Prognosis in Patients with Lyme Carditis

As the case studies in our review illustrate, there is a common clinical trajectory that patients with LC typically follow. Antibody testing and Western blot are used to confirm acute Lyme infection. Patients are often empirically treated with a third-generation cephalosporin, such as ceftriaxone. Symptoms of shortness of breath, fatigue, and pre-syncope tend to correlate with the degree of conduction disease and heart block. Telemetry and serial 12-lead ECG are used to monitor conduction disease. In the absence of treatment, the degree of heart block tends to progressively worsen (i.e., paroxysmal conversion from first- to second-degree Mobitz I heart block, followed by spontaneous conversion to Mobitz II or complete heart block). Once an appropriate antibiotic course is begun, reversion from higher-grade heart block to either first-degree heart block or normal sinus rhythm ensues, usually over the course of days to weeks. Periodic ambulation to test for chronotropic incompetence is also common. Upon the resolution of symptoms, patients are typically transitioned to oral doxycycline as outpatients.

Other diagnostic testing for cardiac complications includes echocardiogram when valvular disease or LV dysfunction is suspected. Laboratory testing of cardiac biomarkers, such as troponin and B-type natriuretic peptide, is used to gauge myocardial insult for evidence of LC. The SILC score can be used to further risk stratify these patients. CMR is pursued to confirm LC, to evaluate for a concomitant infiltrative process, or to aid in the diagnosis, especially in the presence of abnormalities detected by echocardiogram. Major complications present in the form of unstable arrhythmias requiring advanced cardiovascular life support, though their incidence is low. Although rare, for heart blocks that fail to improve with pharmacological treatment, a permanent pacemaker may be indicated.

### 3.5. Longitudinal CMR Findings and Monitoring of Treatment Response

The presence of myocardial edema and scarring detected by CMR has been associated with worse outcomes in patients with myocarditis [19,68]. Patients exhibiting substantial LGE or LV dysfunction may require more intensive monitoring and treatment, potentially influencing decisions regarding the need for pacemaker placement in cases of AV block. CMR is also valuable for monitoring treatment response in LC. Serial imaging can help assess the resolution of myocardial inflammation and the effectiveness of antibiotic therapy, guiding longitudinal clinical decision-making.

Most often, LC and associated conduction abnormalities resolve with targeted antibiotic therapy, with permanent pacemaker implantation a requirement in only a small number of severe cases. Although full clinical resolution can reasonably be expected, in many instances, remnants of past inflammation can be identified on CMR after the acute episode. Of the reviewed cases, nine reported follow-up CMRs (that is, at least one CMR beyond the baseline CMR) (Table 2).

Munk et al. reported the case of a patient with a follow-up CMR four months after initial presentation which showed persistent epimyocardial LGE [43]. In the case reported by Ventejou et al., persistent subclinical myocarditis was identified on CMRs performed three and twelve months after the index presentation [36]. In the case described by Konopka et al., CMR at 12-month follow-up also demonstrated features of inflammation [31].

Three of the cases with longitudinal CMR findings showed near-complete resolution compared with the initial imaging study. Prochnau et al. described almost complete regression of septal edema originally noted in the interventricular septum [50]. Maher et al. reported a case wherein a two-month follow-up CMR showed “significant reduction in the focal areas of late enhancement with only subtle areas of increased signal remaining” [62]. Similarly, in the case described by Naik et al., CMR performed at six weeks follow-up showed a “significantly diminished extent” of LGE in the basal anteroseptal wall [34]. Lastly, there were two cases that demonstrated complete normalization at follow-up. In the case by Koene et al., follow-up imaging revealed no gross myocardial edema or fibrosis at two months [42]. Follow-up imaging in Shah’s case study showed the resolution of two previously noted right ventricular clots [69].

## 4. Limitations and Future Directions

Overall, our comprehensive review highlights the scarcity of published literature on the application of CMR in LC. Our systematic search yielded primarily single case reports, limiting our ability to systematically assess trends or draw robust, data-driven conclusions on the use of CMR in LC. In addition, significant heterogeneity in the level of detail offered in each CMR report challenges the ability to draw nuanced comparisons across studies.

On the other hand, the diversity of included cases underscores the complex nature of LC, with variations in symptomatology, geographic distribution, and CMR tissue patterns. This variety illustrates the multifaceted presentation and clinical progression of LC, emphasizing the need for further longitudinal, population-level studies. Moreover, each case emphasizes the valuable role that CMR can contribute to both the diagnosis and long-term monitoring of LC, providing critical insights into the condition that cannot be captured through traditional diagnostic methods. Furthermore, while the prognostic utility of CMR has been studied extensively in other etiologies of myocarditis—such as viral, eosinophilic, or immune checkpoint inhibitor-associated myocarditis—there remains a need for deeper investigation of phenotypic CMR findings and their associations with long-term outcomes in cases of LC [70,71,72,73,74].

Looking forward, CMR can play a crucial role in diagnosing, prognosticating, and monitoring patients with suspected LC and should be leveraged when available. In addition, with the availability of CMR and advanced cardiovascular imaging specialists concentrated primarily among urban academic medical centers, improved access in rural areas—where LD and LC are more prevalent—could enhance overall care for this condition [75,76].

## 5. Conclusions

LC is an uncommon but potentially fatal and increasingly frequent manifestation of early-disseminated LD. Timely diagnosis requires a high clinical suspicion, coupled with prompt assessment of the extent of cardiac involvement. When available, CMR can play a vital role in the evaluation of myocardial inflammation among patients with suspected LC, reliably distinguishing causes of cardiomyopathy and enhancing clinical decision-making.

## Figures and Tables

**Table 1:** Summary of phenotypic findings in CMR-proven Lyme carditis cases.

Study (*n* = 24)	Patient Demographics and Presentation	Reported CMR Findings
Avitabile, 2015 [56]	A 16-year-old female in the USA presenting with palpitations, and myalgias. No rash, arthralgias, or preceding febrile illness.	Preserved LV function Global edema and hyperemia on T1- and T2-weighted sequences, respectively LGE in the ventricular septum and inferior LV wall in basal/mid-myocardium
Bergler-Klein, 1993 [57]	A 22-year-old male with acute severe precordial pain and upper limb arthralgias; no recollection of erythema or tick bite.	T1- and T2-weighted sequences showed epicardial and myocardial edema, mostly anterolaterally
Constante, 2021 [58]	A 17-year-old male in a rural area in Portugal presenting with cervical pain, odynophagia, and fever.	Preserved global systolic function Focal, subepicardial LGE in LV T2-weighted sequence with LV wall edema
Costa, 2023 [59]	A 26-year-old male in Switzerland presenting with intense chest pain, fever, and palpable inguinal lymphadenopathy.	Non-ischemic areas of enhancement in the LV with edema/inflammation Contractility abnormalities
Cunningham, 2017 [37]	A 12-year-old male in the USA, previously healthy, presenting with cardiac arrest after recent outdoor camping trip.	Early and late gadolinium enhancement of mid-anteroseptal and basal inferior segments of LV
Eslami, 2020 [60]	A 62-year-old male in the USA with 1 week of myalgias, drenching sweats, fever, and chills.	CMR-confirmed myocarditis
Gadela, 2021 [48]	A 21-year-old male in the USA with chest pain and a tick bite a few weeks prior.	Patchy, intramural, myocardial enhancement consistent with myocarditis
Inboriboon, 2010 [61]	A 20-year-old male in the USA with 2 weeks of migratory arthralgias, numerous macular blanching rashes, and a brief episode of palpitations.	T2-weighted sequence showed edema in the septum and anterior wall and LGE in the apical septum, consistent with inflammation and edema involving the AV node
Kaldas, 2024 [41]	A 72-year-old male in the USA; erythema migrans rash. After 20 days of treatment with doxycycline, VT arrest and cardiogenic shock occurred.	Multiple patches of LGE and T2-weighted sequence edema in a mesocardial pattern No evidence of infarct, despite left heart catheterization revealing severe LAD stenosis
Konopka, 2013 [31]	A 41-year-old female in Poland; 4 months of progressive fatigue, exertional dyspnea, and atrial fibrillation.	Mid-wall focus of LGE in the apical region.
Maher, 2012 [62]	A 41-year-old male in Europe with chest pain and recent flu-like illness.	Multiple band-like regions of LGE in the mid-myocardium of the LV wall, sparing the subendocardial layer.
Malik, 2021 [63]	A 22-year-old male in the USA presenting with several days of chest pain and lightheadedness.	Nodular subepicardial LGE in non-vascular distribution
Mehta, 2021 [40]	A 58-year-old male in the USA with myalgias, weakness, fevers, and bilateral facial palsy.	Focal mid-myocardial to epicardial LGE in the basal inferior wall Mild edema in T1- and T2-weighted sequences
Mittal, 2023 [33]	A 67-year-old male with joint pain.	Extensive LGE of multiple myocardial segments Mediastinal lymphadenopathy
Munk, 2007 [43]	A 34-year-old male in Norway; 5-day history of lightheadedness, dyspnea, and chest heaviness on exertion.	Normal LV function Epicardial LGE in the anterolateral wall ∙
Muschart, 2015 [49]	A 13-year-old female in Belgium with malaise and palpitations.	Inferobasal and posterobasal spot compatible with a specific carditis
Naik, 2008 [34]	A 59-year-old male with extreme fatigue and recurrent syncope.	Normal cardiac function Single linear focus of midwall LGE of the basal anteroseptal wall T2-weighted sequence showing subtle edema
Ponniah, 2019 [64]	A 62-year-old male in the USA with ataxia, dysarthria, myalgia, low-grade fever, and shortness of breath.	Subtle LGE in the mid-anterior wall Normal perfusion pattern (ruling out ACS)
Prochnau, 2022 [50]	A 34-year-old in Germany with progressive dyspnea and transient general rash 2 weeks prior.	T2-weighted sequence showing interventricular septal edema No LGE
Rojas-Salvador, 2023 [65]	A 28-year-old male in the USA with fever and fatigue, then rash, lightheadedness, fever, and sinus bradycardia.	Focal LGE in the basal anteroseptal area near the AV node
Schroeter, 2022 [35]	A 37-year-old male in Germany with a history of microscopic polyangiitis on immune suppressive therapy, presenting with severe dyspnea 2° to acute heart failure.	Severely reduced LV ejection fraction Global LV hypokinesia Edema of right and left ventricles No LGE
Tamez, 2011 [66]	A 40-year-old male in Massachusetts; retrosternal chest pain radiating to the left shoulder.	Mild LV systolic dysfunction Hypokinesis of middle and distal lateral segments Epicardial and mid-myocardial LGE of the same segments
Ventejou, 2021 [36]	A 64-year-old male traveling through the USA (treated in France) with erythematous macules and no recollection of a tick bite. Presented to hospital 19 days later with apyrexia, tachycardia, multiple ECM, and facial palsy.	T2-weighted sequence showed myocardial edema Elevated ECV No LGE
Yarema, 2024 [44]	A 51-year-old patient in Ukraine with postural chest pain, shortness of breath, dizziness, headache, several general weakness, and periodic hypertension.	Normal LV function CMR showed signs of myocarditis

Abbreviations: ACS, acute coronary syndrome; AV, atrioventricular; CMR, cardiac magnetic resonance imaging; ECV, extracellular volume; LAD, left anterior descending artery; LGE, late gadolinium enhancement; LV, left ventricular; VT, ventricular tachycardia.

**Table 2:** Phenotypic findings among cases reporting multiple CMRs.

Study (*n* = 9)	Initial CMR Findings	Follow-Up Time	Follow-Up CMR Findings
Cunningham, 2017 [37]	Early and LGE in the midanteroseptal segment and basal inferior segment of the left ventricle.	~3–5 weeks	Near resolution of previously noted early and LGE.
Koene, 2012 [42]	Normal systolic function, no edema or fibrosis.	2 months	Preserved systolic function, still no edema or fibrosis.
Konopka, 2013 [31]	Midwall focus of LGE in apical region.	12 months	Features of past inflammation without progression.
Maher, 2012 [62]	Multiple band-like regions of LGE in the mid-myocardium of the LV wall, sparing the subendocardial layer.	3 months	Reduction in the focal areas of LGE, subtle areas of persistent increased signal.
Munk, 2007 [43]	Normal LV function, epimyocardial LGE in anterolateral wall.	4 months	Persistent epimyocardial LGE.
Naik, 2008 [34]	Linear focus of LGE in midwall region of the basal anteroseptal wall.	6 weeks	Significantly diminished extent of LGE in the basal anteroseptal wall.
Prochnau, 2022 [50]	Interventricular septal edema; no LGE.	Unknown	Near-complete regression of septal edema.
Shah, 2018 [69]	No abnormal LGE; two clots in RV apex.	Unknown	Resolution of clots.
Ventejou, 2021 [36]	Myocardial edema, elevated extracellular volume, no LGE.	3 and 12 months	Persistently elevated ECV and myocardial edema at 3 and 12 months.

Abbreviations: CMR, cardiac magnetic resonance imaging; ECV, extracellular volume; LGE, late gadolinium enhancement; LV, left ventricular; RV, right ventricular.

## Data Availability

No new data were created or analyzed in this study.
